# Rapid identification of bacterial select agents using loop-mediated isothermal amplification

**DOI:** 10.1186/s12879-024-09573-w

**Published:** 2025-01-14

**Authors:** Timothy E. Egbo, Candace D. Blancett, Jackie M. Payne, Christopher P. Stefan, Timothy D. Minogue, John H. Sellers, Jeffrey W. Koehler

**Affiliations:** Diagnostic Systems Division, United States Army Medical Research Institute of Infectious Diseases, Fort Detrick, Maryland, 21702 United States of America

**Keywords:** LAMP, Biothreat agents, Infectious disease, Point of need

## Abstract

**Background:**

Point of need diagnostics provide efficient testing capability for remote or austere locations, decreasing the time to answer by minimizing travel or sample transport requirements. Loop-mediated isothermal amplification (LAMP) is an appealing technology for point-of-need diagnostics due to its rapid analysis time and minimal instrumentation requirements.

**Methods:**

Here, we designed and optimized nine LAMP assays that are sensitive and specific to targeted bacterial select agents including *Bacillus anthracis, Francisella tularensis*, *Yersinia pestis*, and *Brucella* spp. Evaluation of each assay determined preliminary limit of detection (LOD) with LOD confirmed across 60 replicates (≥ 95% positivity rate). Testing across a robust set of strains of the target agent, common DNA agents, and near-neighbors documented sensitivity and specificity for independent assays.

**Results:**

Specifically, all assays were 100% specific and sensitive except for *Y. pestis Caf1* (90% inclusive across *Y. pestis* strains).

**Conclusion:**

Here, we optimized assay turn-around-time, decreasing a standard 60 min traditional polymerase chain reaction (PCR) to 30 min using LAMP with positive results in as little as 5–10 min. Incorporating point of need sample processing and evaluating the potential inhibitory impact of sample matrices such as whole blood and soil would be needed to enable this test system for use on field-forward clinical and environmental sample testing.

**Supplementary Information:**

The online version contains supplementary material available at 10.1186/s12879-024-09573-w.

## Background

*Bacillus anthracis*, *Brucella* spp., *Francisella tularensis*, and *Yersinia pestis* are highly infectious zoonotic bacteria that can cause severe disease including anthrax, brucellosis, tularemia, and plague, respectively. The Centers for Disease Control and Prevention (CDC) classified *B. anthracis*, *F. tularensis*, and *Y. pestis* as “Category A” agents and *Brucella* spp. as a “Category B” agent due to its extreme infectivity and ability to persist causing debilitating infections [1]. Having or being able to quickly develop robust assays is essential for early detection and mitigation in the event of an outbreak.

Real-time PCR is widely used for the identification and confirmation of a wide range of different infectious diseases in a clinical and environmental context. This approach is generally limited to reference laboratories due to equipment costs, training, and biosafety needs. Loop-mediated isothermal amplification (LAMP) is a sensitive and rapid nucleic acid detection technology amenable for use in austere environments. Several isothermal amplification techniques have been reported as alternatives to real-time PCR assays, offering quicker time-to-answer and simplified reaction requirements [2]. Previous studies show the utility of isothermal amplification for diagnostics across a variety of methods to include LAMP, helicase dependent amplification (HDA), nicking enzyme amplification reaction (NEAR), and recombinase polymerase amplification (RPA) [3].

Compared to traditional real-time PCR, LAMP assays have lower instrumentation requirements with no need for temperature cycling. Incorporation of intercalating dyes as the assay signal allows detection of etiologic agents manually, eliminating the requirement of fluorescent readout capability.[4, 5] In addition, LAMP assays achieve faster time-to-answer than PCR with removal of the denaturation step due to intrinsic strand displacement activity from the respective polymerase. In contrast to other PCR-based detection assays, LAMP utilizes a set of four primers to generate the required “loop” for amplification. These additional primers increase the complexity of design algorithms; [6] however, LAMP assays are tolerant of minor sequence variations, and the larger number of primers tends to increase reaction specificity [7]. Thus, implementing a well-characterized design workflow for the creation of isothermal amplification assays would be of great benefit for biological select agent assay development.

Here, we developed and characterized LAMP assays for rapid detection of *B. anthracis*, *Brucella* spp., *F. tularensis*, and *Y. pestis*. Assay development employed primer down-selection, limit of detection (LOD) verification, inclusivity, and exclusivity determination. Using verified assays, we have shown that results can be obtained in as little as two hours from sample acquisition.

## Methods

### Bacterial strains

Bacteria isolates and reference strains were obtained from the Biodefense Reference Material Repository (BRMR) Unified Culture Collection (UCC) maintained at the United States Army Medical Research Institute of Infectious Diseases (USAMRIID). Isolates were propagated on blood agar (trypticase soy agar with 5% sheep blood), or chocolate agar for *F. tularensis*, at 37 °C. All *Y. pestis* plates and cultures were incubated at 28 °C.

### DNA extraction and quantification

Propagated bacteria were mixed into Tryptic Soy Broth (TSB) to create a liquid culture and mixed with TRIzol LS (Thermo Fisher Scientific). DNA was extracted using the Qiagen EZ1 robot with the EZ1 DNA tissue Kit (Qiagen, Valencia, Ca) according to the manufacturer’s directions. Samples were quantified using the Qbit Fluorometer and Qbit DNA HS kit (Thermo Fisher Scientific).

### Primer design

Assays were designed in-house, unless otherwise specified (Table 1). Primers for LAMP assays were designed using the freely available software Primer Explorer v5 (http://primerexplorer.jp/lampv5e/index.html). Primer Explorer requires a minimum of a 200-base sequence for primer design, as most LAMP assays will cover > 150 bases. Conserved regions of species-specific targets were designed in house or selected based on previously designed real-time PCR assays [8]. When multiple assays were output by Primer Explorer, assays that included loop primers were preferentially selected for testing as the loop primers are expected to greatly increase reaction speed. Each LAMP assay requires a minimum of four primers (FIP, BIP, F3, and B3), and can also include up to two loop primers (LF and/or LB). Oligonucleotide primers for LAMP DNA amplification (Table 1) were synthesized and HPLC purified by Thermo Fisher Scientific (Waltham, MA) and Integrated DNA Technologies (Coralville, IA). Primers were hydrated with molecular grade water to 100 µM and stored at − 4^o^C.

**Table 1:** LAMP assay panels

Target organism	Target gene	Primer	Sequence (5’-3’)	Source
*Bacillus anthracis*	dhp73.002 chromosome	FIP	GCATGATTGGCTCTTTTGGGATTATAAGCTTGTAGATGATATTTATGACC	USAMRIID
		BIP	AGAATTTTTGCAAGCATTCAAAGGGAATAAATGCTTTTTGCCATAGC	
		F3	GAGAAGAAATTGTAGGATATAAGGT	
		B3	GTGTGCCATCTATTTTGTGAA	
		LB	GGGCTGGAAAACCTATTGACC	
*Bacillus anthracis*	*Pag* (*pXO1*)	FIP	TCGCAATTGATCATTCACTATCTCTAGCGGTATTTAAACCCATTG	USAMRIID
		BIP	ACTAAATCCTGCAGATACACTCCCACATGGAAATGCAGAAGTG	
		F3	TTGGCATTTAATCTTGCTGTA	
		B3	GTAGGACACATACTAGTGAAGT	
		LF	CAGGGGAAAGAACTTGGGCTG	
		LB	ACCAATATCAAAGAACGACGCA	
*Bacillus anthracis*	*capB* (*pXO2*)	FIP	GCTGTTTCCTCATCAATCCCAAGATGATTACATGGTCTTCCCAGA	[9]
		BIP	CGGATCCAGGAGCAATGAGAATACCATTTACGAAGAACGCAG	
		F3	GCGGATAATTCTAGAATTTCAGAAG	
		B3	ATGTTGATGAGGGATCATTCG	
		LF	ACCGCTAAAGCAAGCGATG	
		LB	CGTTTTGCTGACCAATCTAAGC	
*Burkholderia mallei*	*pal*	FIP	TTCACTGCAAGCGTCAGGCGGCGTTTTATCACAAGCGGAC	USAMRIID
		BIP	ATCTGCCCGTCATCGAAATGCACGATGGAATGGGTCTCACG	
		F3	TGCACCGGTATCAGTCGG	
		B3	GGAAGTCGGGATTGTTCTCG	
		LF	GTTGCCGCGGCCGGGATC	
		LB	CTGGTGATCATGAAAACG	
*Burkholderia pseudomallei*	TTS1_TM2	FIP	CCGCCGATTGCTCGACAGTATATACCCCTGGTCGATTTCGT	USAMRIID
		BIP	CCGGAATCTGGATCACCACCACCACCGGTCAGTATCCGTAAC	
		F3	CTCATCGAGCGAATGACGG	
		B3	GCAAGCTGCAACTGCGC	
		LF	GACGCGTGTCGGCTCTT	
		LB	CCTGGAATGAGACCATGAAGC	
*Brucella* spp.	*omp2a*	FIP	ATCTGCAGGCTGCGCATGACTGATAAGCGACGTTGGCG	USAMRIID
		BIP	GGCAATGAACTTTGCACCACCCCGAACCAGAACTACGGTCAG	
		F3	GTAGGAAACTTCCGGCGTAA	
		B3	CATATTCGTCCGCAGCGAC	
		LF	GGGGCAAGACCGCAGTTA	
*Francisella tularensis*	*FOPA2*	FIP	CCTGCAGCATATGGAGTAAACATAGGCTGGTTTAGGTGAAGGT	USAMRIID
		BIP	GGTTGGGCAAATCTAGCAGGTTCTAGATAGTTCAAACTTAAGACC	
		F3	GTACAACCAATTAGTTGGTAGA	
		B3	GTCAACACTTGCTTGAACAT	
		LB	GCAACAGGTGCTTGGGATG	
*Yersinia pestis*	*3a* chromosome	FIP	CACCCGCGTTATCTCATCCCGTTTTCGAGTAGGGTTAGGTGGGC	[18]
		BIP	CATGGACGTATGGCGGGTCATTTTGTGATGCCGTCCAATGCA	
		F3	ACTACCATCCCCTCAAGGTT	
		B3	GAGGGCGTTTTGGTAGAGAA	
		LF	ACCGCCATGAAATGGACAATG	
*Yersinia pestis*	*caf1* (*pMT1*)	FIP	CCACAAGGTTCTCACCGTTTACCTTCACTACAAAAGTGATTGGCAAGG	USAMRIID
		BIP	GGATGACGTCGTCTTGGCTACGTGCAAGTTTACCGCCTTTGG	
		F3	TCAGGATGGAAATAACCACCAA	
		B3	GTTACGGTTACAGCATCAGTGTA	
		LB	GCAGCCAGGATTTCTTTGTTCGC	

### LAMP reaction and primer down selection

The 10x LAMP primer mix was created by mixing 100 μm primers with molecular grade water to create a mixture containing the final concentration of each primer as follows: 16 µM of FIP, 16 µM of BIP, 2 µM of F3, 2 µM of B3, 4 µM of LF, 4 µM of LB, then stored at -4^o^C. Not all assays contained all 6 primers; a list of primers in each assay can be found in Table 1. LAMP reaction master mix was prepared fresh, immediately before tests were run. Assays were assembled in total reaction volumes of 10 µL containing 5 µL WarmStart 2x LAMP master mix (New England Biolabs, Ipswich, MA), 1 µL LAMP 10x primer mix, 0.2 µL 50x Fluorescent dye (SYBR Green), 2.5 µL DNA sample, and brought up to volume with molecular grade water.

All the LAMP assays were conducted in a LightCycler 480 (Roche Life Science, USA) using 96-well plates (Bio-Rad Laboratories Inc., Hercules, CA). Reactions were amplified with single temperature (65^o^ C) 60 s cycles for 30 cycles with quantification every 60 s. The time to answer was optimized, evaluating the positive and negative controls to determine the optimal run time. The positive/negative cutoff was further refined based on the no template controls (NTC) and was defined as the average of the NTCs plus three times the standard deviation of the NTC mean. Upon receipt, primers were mixed, and assays tested against the target bacterial DNA to remove unsuccessful designs.

### DNA quantification, limit of detection, specificity, and sensitivity determination

To determine the LOD, extracted DNA was quantified using the dsDNA assay for Qubit (Thermo Fisher Scientific, Waltham, MA) and diluted with molecular grade water to create 5-fold dilutions spanning across 100 pg/µL – 0.1 pg/µL. Diluted DNA was run with each assay in triplicate. The preliminary LOD was determined based on 3/3 replicates being positive. To confirm the LOD, 60 replicates of the preliminary LOD were run, and a confirmed LOD was accepted when 95% of the replicates showed a positive signal.

Specificity of each assay was tested against a panel of DNA from 152 common species and specific non-target, closely related strains including *Bacillus* spp. (*n* = 15), *Brucella* spp. (*n* = 5), *Yersinia* spp. (*n* = 15), *Francisella* spp. (*n* = 6), and *Burkholderia* spp. (*n* = 7). Sensitivity was confirmed across several strains of each bacterium: *B. anthracis* (*n* = 15), *Brucella* spp. (*n* = 6), *Y. pestis* (*n* = 26), *F. tularensis* (*n* = 15), and *Burkholderia* spp. [*B. mallei* (*n* = 16) and *B. pseudomallei* (*n* = 26)]. All organisms tested are listed in Supplementary Tables 1 and Supplementary Table 2.

## Results

### Primer design and initial testing

LAMP primers were designed internally or adapted from the literature (Table 1). Both *B. anthracis* and *Y. pestis* contain virulence plasmids. We developed assays to detect the bacterial chromosome for *B. anthracis* (DHP73.002) and the *B. anthracis* plasmids *pXO1* (*pag*) and *pXO2* (*CapB*). Not all *B. anthracis* strains contain *pXO1* and *pXO2*, and the plasmids are not exclusive to *B. anthracis.* The incorporation of all three targets allows for identification and characterization of the *B. anthracis* samples. Similarly, the *Y. pestis* assays target the chromosome (*3a*) and the *Caf1* virulence gene on the *pMT1* plasmid, allowing detection of both the organism as well as the virulence plasmid. Molecular differentiation of *B. mallei* and *B. pseudomallei* can be challenging. Here, assays were designed for *B. mallei* (*pal*) and one for *B. pseudomallei* (TTS1_TM2) for successful differentiation. We utilized detection of *omp2a* to identify *Brucella* spp. as speciation without sequencing is also challenging.

### Sensitivity and specificity

**Fig. 1:**
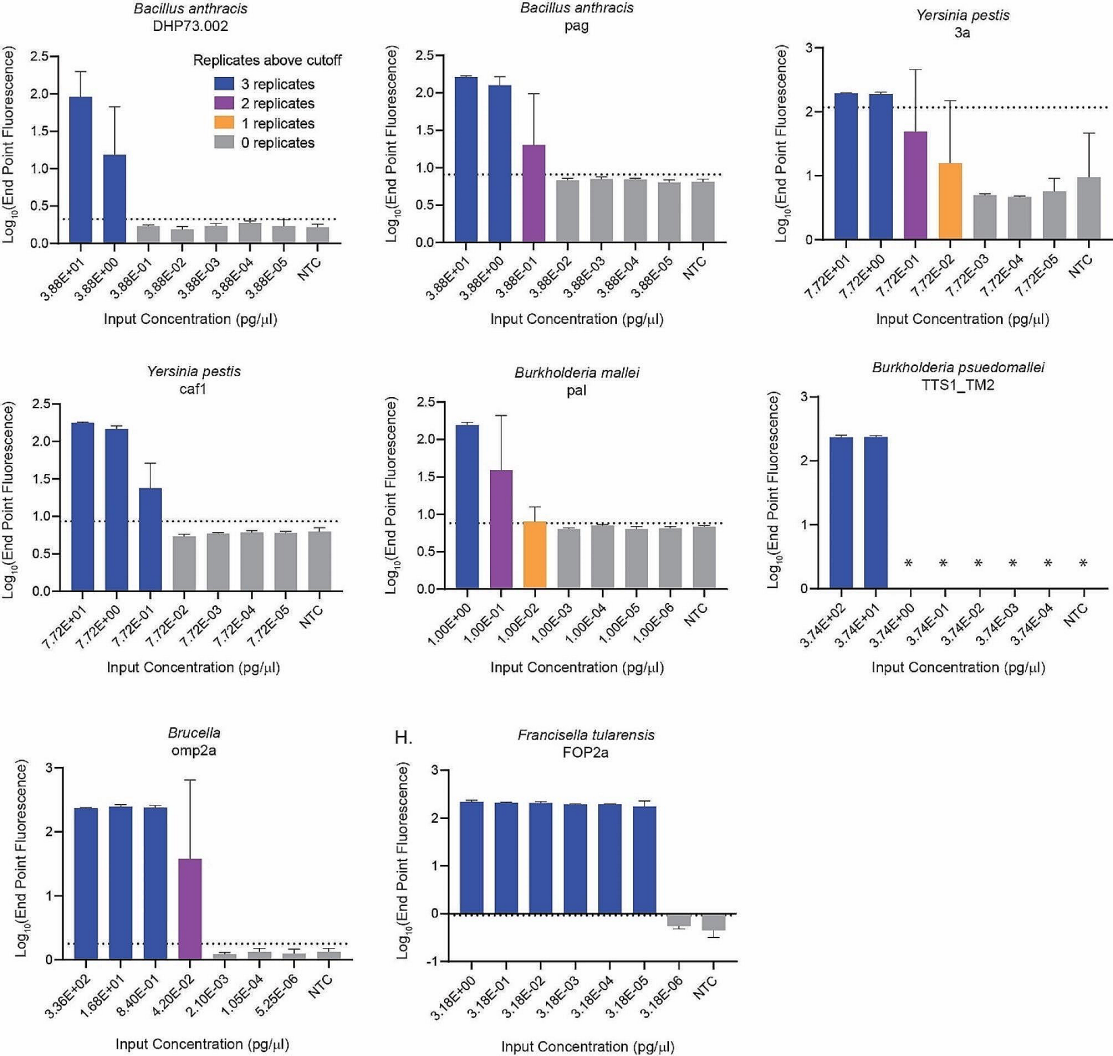
Preliminary limits of detection for LAMP assays. Extracted nucleic acid was serially diluted and tested against organism specific LAMP assays. Bars represent the average log_10_ end point fluorescence 30 min post experiment initiation. Error bars represent standard deviation of three technical replicates, and bar colors indicate the number of replicates above the cutoff value. Cutoff value was calculated using the average plus three times the standard deviation of NTC samples 30 min post experiment initiation. Asterisks represent samples where background fluorescence was negative

Preliminary limits of detection were determined by serially diluting organism nucleic acid and testing with the respective assay (Fig. 1). All assays detected the targeted organism not lower than 1pg/ µL of DNA. The preliminary LODs were confirmed by repeating 60 replicates at the preliminary LOD and increasing the nucleic acid concentration until reaching 95% positivity (58/60 replicates testing positive). Confirmed LODs ranged from 1 to 10 pg/µL of DNA (Table 2).

**Table 2:** Assay metrics

Target organism	Target gene	Preliminary LOD (pg/ul)	Confirmed LOD (pg/ul)	Replicates	NTC Mean (EPF)	Mean (EPF)	Standard Deviation (EPF)	Coefficient of Variance (EPF)
*Bacillus anthracis*	DHP73.002	3.88	10	60/60	9.32	215.7	38.97	18.06%
*Bacillus anthracis*	*pag*	3.88	2	60/60	39.74	216.6	28.03	12.94%
*Yersinia pestis*	*3a*	7.72	5	60/60	17.96	196.1	36.9	18.82%
*Yersinia pestis*	*caf1*	0.772	2	60/60	20.22	169.2	27.7	16.37%
*Burkholderia mallei*	*pal*	1	1	60/60	31.15	199.2	15.99	8.028%
*Burkholderia pseudomallei*	TTS1_TM2	37.4	5	59/60	7.04	180.9	48.13	26.61%
*Brucella* spp.	*omp2a*	0.336	3	60/60	12.44	217	16.71	7.699%
*Francisella tularensis*	*FOP2a*	0.0318	1	60/60	9.66	188.2	17.62	9.364%

Each LAMP assay was tested against a panel of bacterial species the assays should detect (Supplementary Table 1). All strains of *B. mallei* (*n* = 16), *B. pseudomallei* (*n* = 17), *Brucella* spp. (*n* = 6), and *F. tularensis* (*n* = 15) were detected by the respective assay. The three *B. anthracis* LAMP assays detected all 13 strains of *B. anthracis* tested except for *B. anthracis* Sterne. *B. anthracis* DHP73.002 and *pag* detected all 13 strains while *CapB* did not detect *B. anthracis* Sterne. Of note, *B. anthracis* Sterne does not contain the plasmid pXO2 which contains *CapB* while the *pag* assay detects pXO1 plasmid which *B. anthracis* Sterne contains. The results for the *Y. pestis* assays were variable with the *Y. pestis 3a* assay detecting 26 of the 27 strains tested and the *Y. pestis Caf1* assay detecting 23 of the 27 strains. With the two assay results taken together, all *Y. pestis* strains were detected by at least one LAMP assay.

Expanded exclusivity testing was conducted to demonstrate the specificity of the LAMP assays (Supplementary Table 2). Each assay was tested against our standard bacterial exclusivity panel of 46 different bacteria. All assays tested negative for each organism on the panel except for the assays targeting the specific species on the panel. Focused exclusivity panels were tested were evaluated with the specific assays (Supplementary Table 2). For example, the *B. anthracis* assays did not detect the 15 different non-anthracis *Bacillus* strains tested. Specificity evaluations of the *Y. pestis 3a* and *Caf1* assays, all eleven closely related organisms in the genus *Yersinia* were tested and showed discrimination from other *Yersinia* spp. except one of the three replicates that tested positive for *Yersinia enterocolitica* and *Yersinia pseudotuberculosis*.

## Discussion

Isothermal amplification assays such as LAMP are an attractive method for rapid and portable detection of infectious diseases from clinical and environmental samples. The method provides an alternative to real-time PCR which can be limited in testing locations and mobility due to equipment requirements. Here, we developed and characterized a panel of LAMP assays for the detection of multiple bacterial threat pathogens.

Reliable detection of pathogenic *Y. pestis* is challenging due to the variable presence of three virulence plasmids in natural isolates [8]. Consequently, we designed our *Y. pestis* assays to target *Caf1* (a major virulence factor found on *pMT1* plasmid). As previously reported, the *3a* assay targeting the chromosomal sequence provided a broad *Y. pestis* assay since some isolates of *Y. pestis* lack *Caf1.*[8] The *Y. pestis 3a* assay described here had improved sensitivity and performance (four times lower LOD) than other reported assays targeting *3a* [8]. A lower LOD improves accuracy when analyzing samples with a very low concentration of the agent. We expect our assays to maintain similar LODs when applied to clinical samples as LAMP assays have been shown to maintain sensitivity in environmental and clinical samples with low target copies per reaction [10–12].

There is > 90% DNA sequence homology between *Y. pestis* and *Y. pseudotuberculosis*, which commonly leads to cross reactivity due to shared *pYV* or *pCD1* plasmid associated with pathogenesis [13]. Prior research efforts describe the detection of *Y. pestis* using LAMP and other diagnostic techniques [8, 14, 15]. Other reported LAMP assays target one virulence plasmid, while our *Caf1* assay targets all three plasmids, enabling our *Caf1* assay to pick up more *Y. pestis* strains. The results show the primers designed in this study have a high level of sensitivity for *Y. pestis* with the *Caf1* assay being less specific to *Y. pestis* than the *3a* assay. Because of the genetic distinction between *Caf1* and *3a* isolates of *Y. pestis*, a single target LAMP assay in itself is not sufficient for the detection of *Y. pestis*. Therefore, based on the promising data from our study, a multiplexed LAMP detection method for *Caf1* and *3a* could be developed for reliable identification of *Y. pestis* in the future.

A limiting factor in developing rapid detection assays for biothreat agents is the inability to test assay specificity among closely related species [12–14]. We addressed this by employing a robust panel of DNA from closely related species to show a high level of specificity among all the assays analyzed. We evaluated a *capB* assay targeting *B. anthracis* assays, but we had sub-optimal performance compared to the *pag* and *dhp* assays (data not shown). Other studies showed similar non-specificity of *capB*[12, 15, 16] due to the close relationship of *B. anthracis* with other members of the cluster strains known as “senus lato.”[12, 17].

Integrating sample processing and reagent lyophilization into the test system will expand the utility of these assays and technology. We are currently evaluating various point of need sample processing methods for extraction efficiency and pathogen inactivation. Inhibitors found in complex matrices such as whole blood or soil samples can interfere with molecular assays, and additional testing using these matrices are needed for advanced assay characterization. Incorporating these methods will allow for a full sample to answer validation study needed for fielding as a confident testing capability.

## Conclusions

In summary, we designed sensitive and specific isothermal amplification assays to detect five biological select agents with shorter turnaround time (within 30 min) compared to traditional PCR. Future research will focus on decreasing the assay limit of detection, assessment in complex matrices, and deployment of these assays in a fieldable environment using a handheld portable device suitable for a rapid emergency response to an outbreak.

## Electronic supplementary material

Below is the link to the electronic supplementary material.

Supplementary Material 1

## Data Availability

The datasets used and/or analyzed during the current study are available from the corresponding author on reasonable request.

## Abbreviations

BRMR: Biodefense Reference Material Repository
CDC: Centers for Disease Control and Prevention
HDA: Helicase dependent amplification
LAMP: Loop mediated isothermal amplification
LOD: Limit of detection
NEAR: Nicking enzyme amplification reaction
RPA: Recombinase polymerase amplification
USAMRIID: United States Army Medical Research Institute of Infectious Diseases
